# Comparative effectiveness of two methods for inducing osteoarthritis in a novel animal model, the Diannan small-ear pig

**DOI:** 10.1186/s13018-021-02734-6

**Published:** 2021-10-14

**Authors:** Di Jia, Ruixian Zhang, Yinghong He, Guofeng Cai, Jiali Zheng, Yuye Yang, Yanlin Li

**Affiliations:** 1 Department of Sports Medicine, The First Affiliated Hospital, Kunming Medical University, #295, Road Xichang, District Xishan, Kunming, 650000 Yunan China; 2 Department of Environment-Related Health, Center for Disease Control and Prevention of Yunnan Province, Kunming, 650034 China; 3 Department of Reproductive Medicine, Kunming Angel Woman’s and Children’s Hospital, Kunming, 650000 China

**Keywords:** Osteoarthritis, Cartilage, Degeneration, Mini pig, Animal model, Inflammation

## Abstract

**Background:**

Varieties of animals were used to study osteoarthritis pathogenesis. The Diannan small-ear pig, which is native to Yunnan, China, is thought to have an articular anatomy similar to that of humans and is more likely to be a source of pathological tissues than other animals. The aim of this study was to determine whether this animal can serve as a more effective osteoarthritis model and explore the role of SDF-1/CXCR4 signaling pathway in the development of Osteoarthritis in animals.

**Methods:**

Twenty-seven adult pigs were randomly divided into three groups and underwent the Hulth procedure, papain articular injection, and conventional breeding. After 4, 8, and 12 weeks, cartilage tissues from knee joint were extracted for general and histological observation, immunofluorescence, and biochemical analysis. Synovium was taken out for stromal cell-derived factor-1 analysis.

**Results:**

Histopathological observation showed obvious cartilage loss in two experimental groups, this cartilage loss was more severe in the chemical groups. Synovial stromal cell-derived factor1 levels increased over time in all groups. mRNA and protein levels of matrix metalloproteinase-3 were much higher in the chemical groups than in the other groups, whereas levels of collagen type II and aggrecan were significantly lower in the chemical groups than in the other groups. Immunofluorescence assays of collagen type II revealed an apparent reduction in this marker in the chemical groups compared with the other groups.

**Conclusions:**

These results indicated that the Diannan small-ear pig can be used as an effective osteoarthritis model. In addition, it is much more convenient and much faster to induce osteoarthritis by intra-articular injection of papain, which is a method worthy of being promoted.

## Background

Osteoarthritis (OA) is a multifactorial disease characterized on the basis of structural changes in articular cartilage and periarticular bone [1–4]. Stromal cell-derived factor-1 (SDF-1) was identified as a strong inflammatory cytokine existing in the synovium, and its specific receptor C-X-C chemokine receptor type 4 (CXCR4) locates on the surface of chondrocytes [5]. The binding of SDF-1 and CXCR4 can lead to a cascade of matrix metalloproteinase (MMP) release and collagen (mostly collagen type II, Col II) and proteoglycan (mostly aggrecan, ACAN) degradation, hence exacerbating and accelerating the OA process [6].

Small animals have been selected to study OA and to determine disease-specific biomarkers for better OA knowledge and therapy [7, 8]. Unfortunately, it is difficult to obtain enough pathological tissues in small animals (mouse, rat, and rabbit), particularly on an eroded cartilage surface. However, large animals (horse and bovid) are difficult to manage and breed [9, 10]. Diannan small-ear pig is a kind of mini pig produced in Yunnan with the average maturation age of 8 months. The pig is easy to manage and has strong resistance to mosquitoes and parasites compared with other pigs. The pig was used because it has similarity to human in terms of anatomy, physiology, and pathology, which shows an advantage in disease models and pharmaceutical research [11].

Commonly methods were used to induce OA, including surgery and chemistry. Among surgical methods, the Hulth technique is deemed to be rapid and effective, consisting of Anterior Cruciate Ligament (ACL), Posterior Cruciate Ligament (PCL), Medial Collateral Ligament (MCL), and Medial Meniscus (MM) transection [12]. Another approach is intra-articular injection of chemicals (mostly papain). Papain is a proteolytic enzyme which results in a series of inflammatory mediators being produced, and which causes cartilage breakdown [13]. Although various approaches can induce OA, the best one to mimic the pathological process while still involving a short modeling time remains controversial.

This study addressed the issues of obtaining well-documented evidence for whether the Diannan small-ear pig can be regarded as an effective OA model and whether we can identify a better way of OA modeling. At the same time, the key role of SDF-1/CXCR4 signaling pathway in the pathological process of OA in Diannan small-ear pig was explored.

## Methods

### Experimental models

Animal studies were approved by the Institutional Animal Care and Use Committee of Kunming Medical University. Twenty-seven Diannan small-ear pigs (average age of 9 months old, 20 males and 7 females, average weight of 19.8 ± 2.5 kg) were obtained from the Animal Laboratory of Kunming Medical University, none of them were subjected to other experimental procedures. They were randomly divided into three groups: operation group, chemical group, and control group. Operation groups underwent the Hulth procedure in right knee joints. Intravenous injection of pentobarbital sodium at the ear margin of 20–40 mg/kg was used for anesthesia induction followed by 5–40 mg/kg/hour for maintenance of anaesthesia. In brief, the right joint was incised with a medial approach at the patellar tendon, followed by lateral dislocation of the patella, and the articular cavity was exposed. Finally, the capsule and skin were closed with sutures. Chemical groups received 4% papain intra-articular injection in the medial tibiofemoral joint gap of the right knee for three consecutive days. The control group left with conventional breeding. Animals were housed individually in well ventilated standard pig cages with daily feeding in in an animal facility with a 12-h light and 12-h dark cycle and room temperature. In order to mimic the pathological process of OA and to establish OA model in the short term, pigs were released from the cages and driven to run back and forth on the 30-m-long road for 10–15 min every three days. Three pigs in each group were sacrificed at 4, 8, and 12 weeks after surgery, and their tissues were harvested for further analysis. All pigs were eventually euthanized with 100 mg/kg of pentobarbital sodium intravenously injected at the ear margin.

### Sample collection

Following the experimental procedure, cartilage specimens were taken out from weight-bearing areas of medial femoral condyles to use for biochemical analysis. Full-thickness cartilage tissues, including subchondral bone, were collected to undergo histological staining. Hyperemic synovial tissues were extracted to detect SDF-1 levels.

### Macroscopic and histological observations

Following the incision of the knee, cartilage degeneration and synovial inflammation were assessed by general observation. For histological detection, osteochondral tissues (6 × 6 mm) from weight-bearing regions were collected and prepared for paraffin embedding. Then specimens were sectioned in the coronal plane, cut into 5-μm-thick slices, and stained with Hematoxylin (H&E) and Safranin O fast green to observe the cartilage degeneration and distribution of chondrocytes. Images were acquired using a light microscope. Four slices from each of the cartilage specimens were selected and examined, and a modified Mankin score was calculated to evaluate the cartilage damage. For fluorescence staining, 5-μm-thick slices were incubated with monoclonal mouse-anti-human collagen II antibodies (1:50 dilution, LifeSpan BioSciences, Inc, Seattle, WA, USA) at 4 °C overnight, followed by incubation with affinity-purified antibody DyLight 488 labeled goat anti-mouse immunoglobulin G (IgG) (H + L) (1:1000, Abcam, Cambridge, UK) at room temperature for 1 h. Nuclei were counterstained with DAPI. The intensity of collagen II-positive staining was analyzed with integrated optical density (IOD) using Image-Proplus 6.0 software. The intensity of positive staining was analyzed by average optical density (OD). OD is defined as integrated optical density (IOD) per stained area (μm^2^) (IOD/area) for positive staining.

### ELISA

10 mg synovium was cut into pieces and homogenized in an ice bath. After centrifugation for 5 min at 4 °C, supernatant was extracted and used for the following test. SDF-1 levels in the synovium of each group were analyzed using an ELISA kit (R&D Systems, Minneapolis, MN, USA) according to the manufacturer's instructions. OD values in all plates were calculated at the 450 nm wavelength using a microplate reader, and SDF-1 contents were determined based on a regression equation of standard concentrations and corresponding OD values.

### Real-time quantitative polymerase chain reaction assay

Total RNA was extracted from cartilage tissues and purified with TRIzol (Invitrogen, Life Technologies, Paisley, UK). The resulting cDNA was used as the template for PCR amplification. Real-time PCR was performed using SYBR green dye with the following parameters: an initial denaturation step at 95 °C for 10 min; 40 cycles at 95 °C for 15 s, 60 °C for 20 s, and 72 °C for 30 s. The mRNA levels of the internal reference GAPDH were determined for each sample to quantify the relative mRNA expression levels of MMP-3, Col II, and ACAN. Primer sequences for genes are listed in Table 1. Ct values were normalized against GAPDH, and relative expressions of each gene were calculated as 2^−ΔΔCt^.

**Table 1:** Primers sequences for RT-qPCR assay

Gene Symbols	Primer sequences	Length
GAPDH	F 5′-TCTGGCAAAGTGGACATT-3′	84 bp
	R 5′-GGTGGAATCATACTGGAACA-3′	
MMP3	F 5′-AAGTGTTATTGATTCTACCATTG-3′	98 bp
	R 5′-TTATGTCAGCCTCTCCTT-3′	
ColII	F 5′-AGCAAGAGCAAGGACAAG-3′	96 bp
	R 5′-AGTGTTAGGAGCCAGGTT-3′	
ACAN	F 5′-TAGAAGGAAGAGGAACCAT-3′	75 bp
	R 5′-TAATGTCCAACTCACTGAAG-3′	

F forward, R reverse

### Western blotting

Cartilage tissues (100 mg) in liquid nitrogen were homogenized and added to 400-μl RIPA buffer containing 50 μl protease inhibitor. After 20 min’ standing in an ice bath, the homogenate was centrifuged (12,000 RPM at 4 °C) for 10 min. The supernatant was collected, and the protein concentration was quantified using a BCA Protein Assay Kit (Pierce, Rockford, USA). The total protein (80 μg) was electrophoresed in SDS-PAGE and transferred to a polyvinylidene difluoride (PVDF) membrane (Millipore, Bedford, MA, USA). The PVDF membrane was blocked and gently shaken with 5% Bovine Serum Albumin (BSA) for 1.5 h at room temperature and was then stored overnight at 4 °C with the following antibodies: mouse monoclonal antibody against human collagen II (1:1000 dilution, LifeSpan BioSciences Inc, Seattle, USA), rabbit polyclonal antibody against human ACAN (1:1000 dilution, LifeSpan BioSciences Inc, Seattle, WA, USA), mouse monoclonal antibody against human MMP-3 (1:1000 dilution, LifeSpan BioSciences Inc, Seattle, USA). Horseradish peroxidase-conjugated anti-rabbit or anti-mouse (1:2000 dilution, Cell Signaling Technology, Danvers, MA, USA) were used as secondary antibodies, and the expression of GAPDH (1:1000 dilution, Cell Signaling Technology, Danvers, MA, USA) was used as the internal reference for each group. Protein bands were quantified by Image J.

### Statistical analysis

All quantitative data were analyzed with SPSS 18.0 (SPSS, Inc. Chicago, IL, USA) and described as mean ± standard deviation. Statistical analysis was performed using one-way analysis of variance (ANOVA) with the least-significant difference correction to determine differences between groups, and *P* values < 0.05 were considered statistically significant.

## Results

### Characterization of cartilage degeneration in Diannan small-ear pigs

**Fig. 1:**
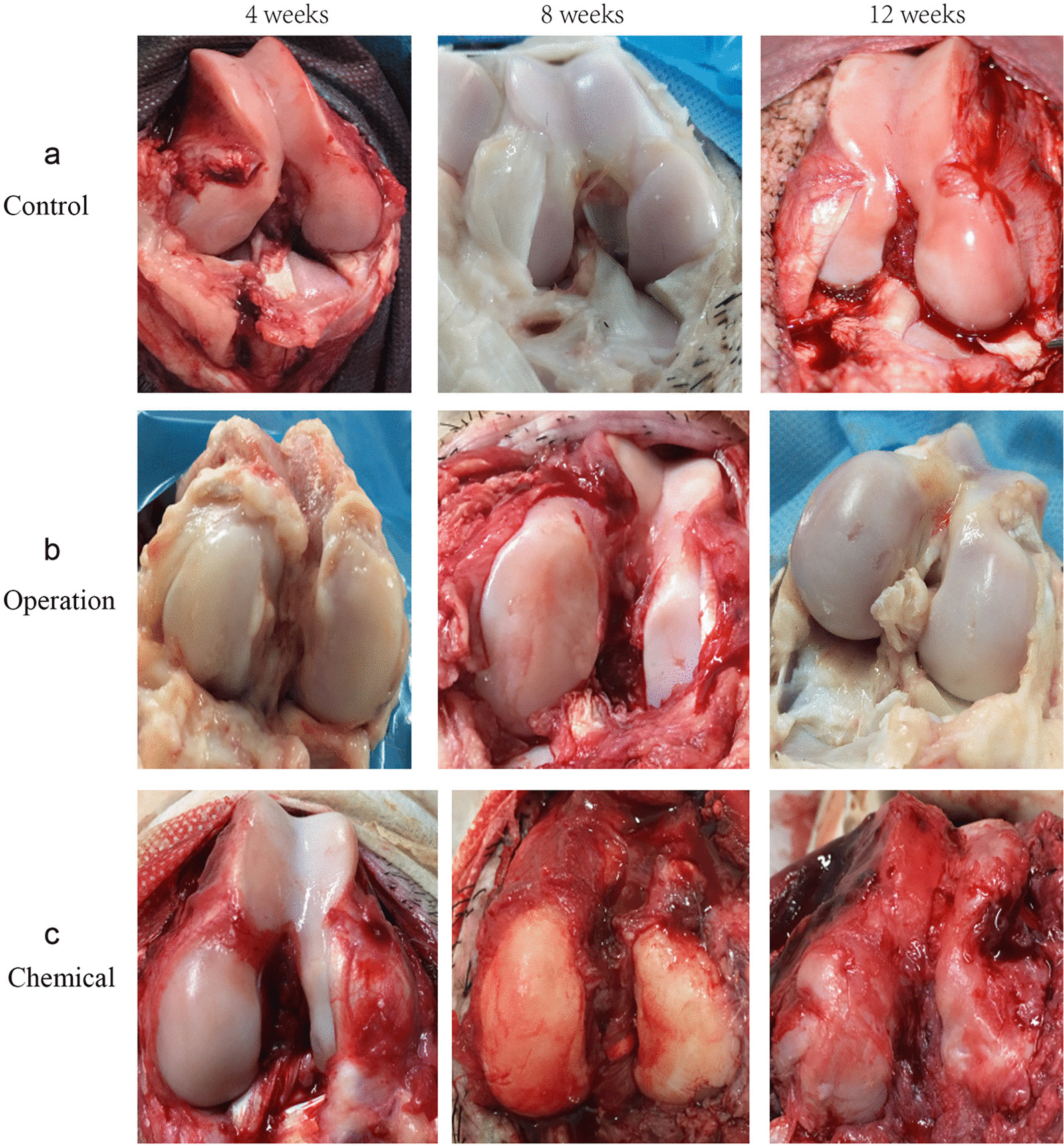
Macroscopic view of pig articular cartilages of the different groups at the experimental time periods indicated. **a** Pigs left untreated. **b** Pigs that underwent the Hulth technique. c Pigs that received papain injection

There were no hallmarks of OA in the articular surfaces in the control group at 4 and 8 weeks, mild shade was detected at 12 weeks (Fig. 1a). At 4 weeks, dulling was observed in the operation and chemical group. At 8 weeks, articular surfaces of the two experimental groups showed macroscopic irregularity. The synovial hyperemia and roughness were more prominent in the chemical group. Dramatic cartilage lesions and cartilage loss were detected in the operation and chemical groups at 12 weeks. Furthermore, the chemical group exhibited more serious cartilage damage and synovial inflammation (Fig. 1b, c).

**Fig. 2:**
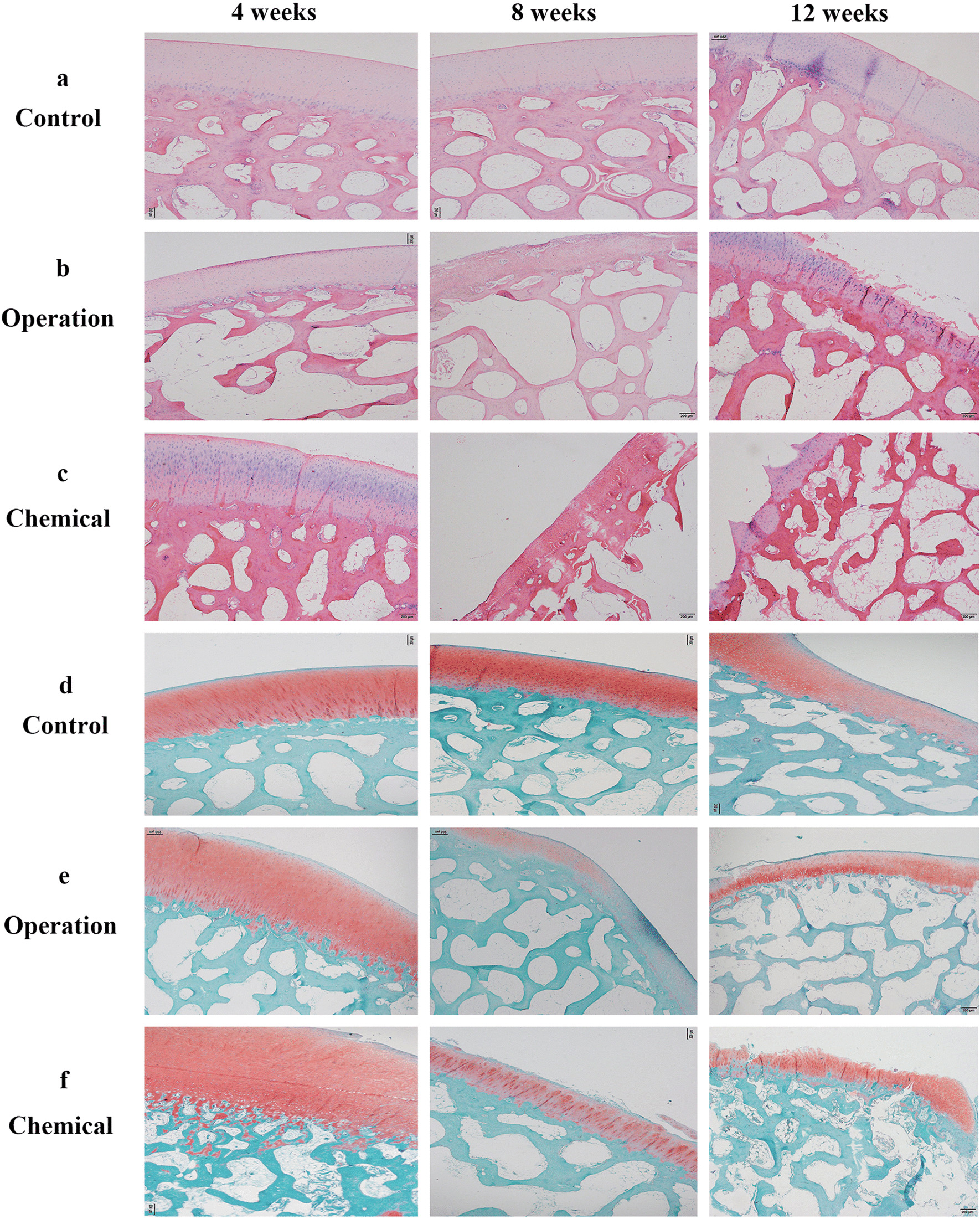
Effects of cartilage degeneration on different methods by histopathological staining (**a**–**c**: HE staining, **d**–**f**: safranin O fast green staining). Scale bar: 200 μm. **a**, **d** Representative cartilage tissues extracted from control groups at 4, 8, and 12 weeks. **b**, **e** Representative cartilage tissues extracted from operation groups at 4, 8, and 12 weeks. **c**, **f** Representative cartilage tissues extracted from chemical groups at 4, 8, and 12 weeks

For histological staining, normal manifestations of cartilage were seen in control groups at 4 and 8 weeks, whereas slight roughness appeared at 12 weeks (Fig. 2a, d). Uneven chondrocyte distributions in the cartilage layer were detected in the operation group, and a rough surface of cartilage was detected in the chemical group at 4 weeks (Fig. 2b, c, e, f). At 8 weeks, several cartilage lesions and irregular chondrocyte distributions appeared in the operation and chemical groups, and partial subchondral bone damage was observed in the chemical group (Fig. 2b, c, e, d, f). Compared with other groups, Safranin O fast green stained weakly in the operation group at this time (Fig. 2e). Numbers of chondrocytes fell in both experimental groups at 12 weeks, and cracking was increased in the operation group. Meanwhile, extensive chondral fracture and subchondral bone exposure occurred in the chemical group (Fig. 2b, c, e, f). By comparing the Mankin score in each group, we found that there were significant differences among the three groups, except between the 4-week and 8-week time points in the control group (Table 2).

**Table 2:** Comparison of modified Mankin scores within groups and followed times, respectively (‾χ ± s, n = 3)

Group	Time (weeks)			*p* value
	4	8	12	
Control	**0**	**0**	2.25 ± 0.50	** < 0.01**
Operation	3.25 ± 0.96^*^	7.00 ± 0.82^*^	10.00 ± 1.41^*^	** < 0.01**
Chemical	5.00 ± 1.15^#^	6.75 ± 1.26^#^	12.75 ± 0.50^#^	** < 0.01**
*p* value	** < 0.01**	** < 0.01**	** < 0.01**	**–**

“^*^” indicates significant difference between operation and control groups after the same weeks, while “^#^” between chemical and operation groups

### Cartilage degeneration is associated with expression of Col II

**Fig. 3:**
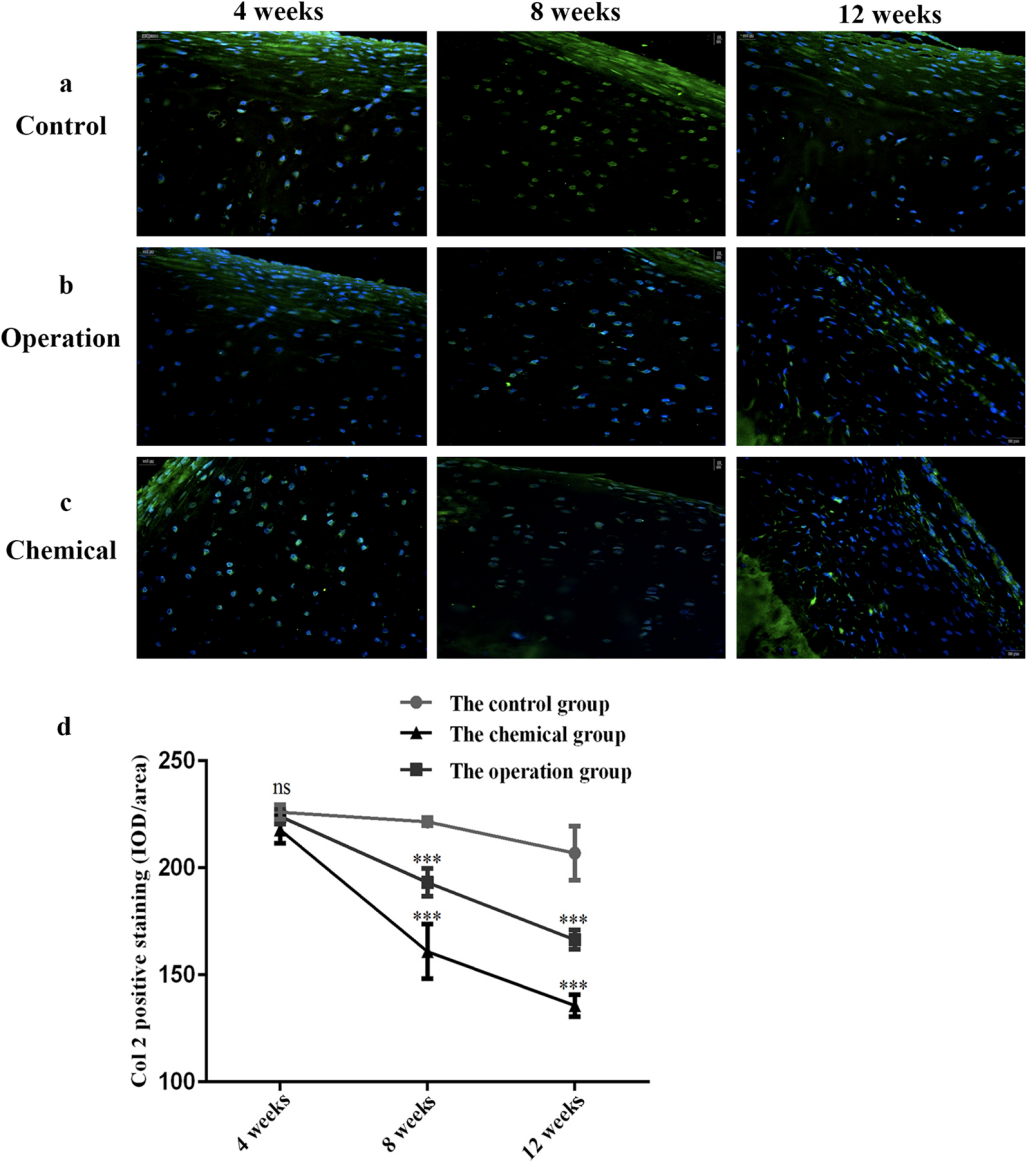
Expression of Col II in the cartilage. Scale bar: 20 μm. **a** Col II expression of control groups at 4, 8, and 12 weeks. **b** Col II expression of operation groups at 4, 8, and 12 weeks. **c** Col II expression of chemical groups at 4, 8, and 12 weeks. **d** IOD/area values of Col II. ***p < 0.001; ns indicated that there was no statistical difference compared with the corresponding group. Col 2, collagen type two

Green fluorescence of col II was distributed in the superficial and subchondral areas of cartilage in all groups. It was also detected in the cytoplasm and cytomembrane, but not in the nucleus (Fig. 3a–c). Two experimental groups exhibited a significant reduction of Col II (Fig. 3a–c). The IOD/area values of two experimental groups were significantly lower than those in controls. Among the three groups, they were lowest in the chemical groups, which was consistent with our biochemical analysis (Fig. 3d).

### Increased SDF-1 levels in the synovium

**Fig. 4:**
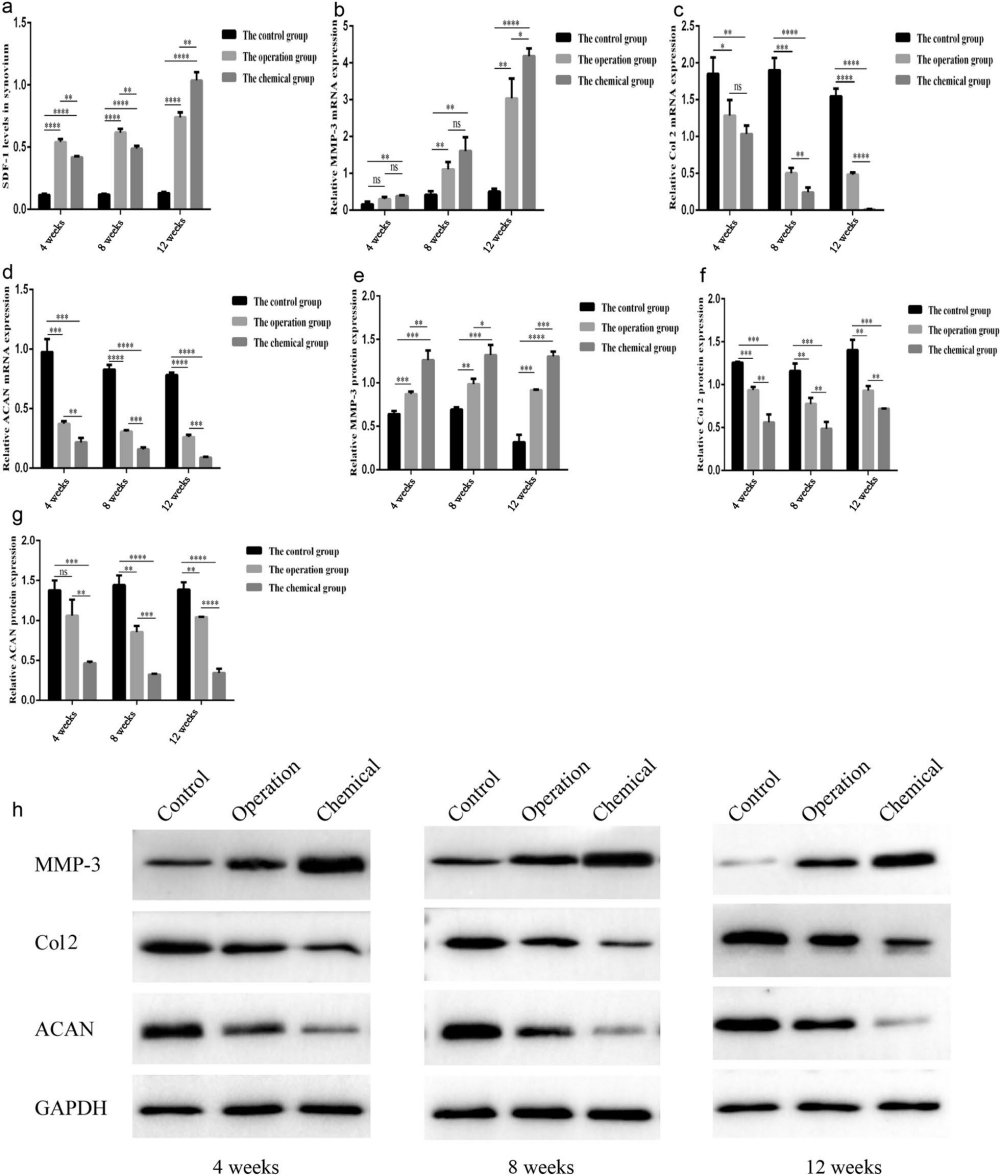
Effect of OA related genes at mRNA and protein levels. **a** levels of SDF-1 in the synovium of pigs measured by ELISA. **b**, **c,** and **d** mRNA levels of MMP-3, ColII, and ACAN. **e**, **f**, and **g** protein levels of MMP-3, ColII, and ACAN. (h)protein electrophoretogram of MMP-3, ColII, ACAN, and GAPDH. *p < 0.5; **p < 0.01; ***p < 0.001; ****p < 0.0001; ns indicated that there was no statistical difference compared with the corresponding group. SDF-1, stromal cell derived factor-1; MMP-3, matrix metalloproteinase 3; Col 2, collagen type two; ACAN, aggrecan; GAPDH, glyceraldehyde phosphate dehydrogenase

Levels of SDF-1 in both experimental groups evidently increased compared to the controls (*P* < 0.05). Over time, SDF-1 levels in the three groups gradually increased. Comparisons among three groups showed that there were statistical differences (*P* < 0.05) (Fig. 4a).

### Expression profiles of MMP-3, COL II, and ACAN associated with ECM degeneration on the cartilage tissues

MRNA levels of MMP-3 in the two experimental groups increased over time, and they were higher than those in the control groups (*P* < 0.05) (Fig. 4b). mRNA levels of Col II and ACAN in the two experimental groups decreased as time prolonged, and they were lower than those in the controls (*P* < 0.05) (Fig. 4c, d). For MMP-3, Col II, and ACAN mRNA levels, two comparisons between the three groups were statistically significant (*P* < 0.05). As shown by western blotting, expression patterns of these genes were similar to those shown by RT-qPCR analysis (Fig. 4e–h).

## Discussion

The Hulth technique is a surgically induced method that transects key structures in the joint, causing joint instability. Papain is a proteolytic enzyme that results in a series of inflammatory mediators being produced and causes cartilage breakdown [14, 15]. Papain injection is strongly recommended because it provides an easy approach and less trauma. Moreover, the chemical method leads to a more severe and rapid OA process than surgery in terms of the general performance and experimental analysis. These results may contribute to better ways of inducing OA. Interestingly, slight roughness were observed in the control groups at 12 weeks. The reason for the injuries in the controls may be attributed to an artificially increased activity, which improved the frequency of joint surface friction, and it is consistent with the pathogenesis of knee osteoarthritis [16].

OA is a progressive disease accompanied by aseptic inflammation [17, 18]. SDF-1 is considered to be a proinflammatory factor released from synovial tissues [19]. SDF-1 was found to be obviously increased in osteoarthritic synovium, and the level in serum dramatically reduced after synovectomy, which strongly indicated a key role of SDF-1 in OA pathogenesis [20, 21]. In our previous study, we showed that SDF-1 levels in osteoarthritic guinea pigs’ serum increased with time, and these levels positively correlated with the cartilage degeneration. The small peptide named T140 was found to be a complete CXCR4 inhibitor and can totally block the SDF-1/CXCR4 signaling pathway, and was thought to have potential in the treatment of OA. On this basis, T140 prevents cartilage from deteriorating and significantly reduces SDF-1 levels by blocking the SDF-1/CXCR4 axis [22]. These results indicated that SDF-1 was closely linked with OA and can be regarded as a biomarker. In this study, a dramatic elevation of SDF-1 was found in the two experimental groups over time, and the highest level was found in the chemical group at 12 weeks. In combination with our further histological and biochemical analyses, higher levels of SDF-1 were in line with more serious cartilage damage. This indicated that SDF-1 can serve as a sign of OA in pigs, and the expression may be positively related to the severity of OA.

Articular cartilage is mainly made up of extracellular matrix (ECM), which is rich in Col II and ACAN. Col II and ACAN were considered as protective factors and play important roles in maintaining the elasticity and hardness of cartilage [23]. Activation of catabolic enzymes such as MMPs, IL-1β, or trauma leads to loss and degradation of Col II and ACAN, in turn which are identified as main pathological characteristics of OA [24]. MMP is a family of proteolytic enzymes which promotes the degradation of ECM [25, 26]. Indeed, MMP-3 has been identified in OA synovium and synovia, and its expression levels are regarded to be relative to OA severity and SDF-1 release [15, 22, 27]. Following the release of MMPs, collagens, and matrix proteins such as ACAN degrade, the OA pathological process accelerates [28]. Our previous studies have determined that levels of col II and ACAN are notably reduced in a spontaneous OA guinea pig model, and they reversibly increased as a consequence of T140 treatment [22]. As displayed in histological staining, deteriorating changes were seen in this study, which showed that OA changes were time dependent in both methods. Cartilage surfaces and subchondral bones were increasingly destroyed, and the fluorescence intensity of Col II decreased in both methods. These results were consistent with early studies [17]. Subchondral bone is a mechanical support of overlying cartilage and plays a critical role in bone modeling and remodeling. Lesions of subchondral bone reflect serious cartilage damage [5]. At 12 weeks, subchondral bone of pigs in two experimental groups was visibly destroyed, especially in the chemical group, which means that late OA appeared at this time; papain injection brought about more severe changes. Furthermore, both RT-qPCR and Western blotting analysis showed up-regulation of MMP-3 in experimental groups compared with controls, and levels increased in the later period. However, levels of Col II and ACAN in experimental groups were lower than in control groups and gradually decreased with time. These results suggested that these markers changed in our OA models, and the variation trends were similar to those in other small animals. Also, both mRNA and protein levels of MMP-3 were found to be highest, and levels of Col II and ACAN were lowest in the chemical groups at 12 weeks. This was in accordance with our histological observations. Another interesting discovery in our study is that SDF-1 notably increased in the synovium of OA pigs no matter which method we used, which suggested that SDF-1 plays key roles in OA development. This finding increases our reasons to be persuaded that inhibition of SDF-1 may be an effective strategy for OA therapy and worthwhile to investigate further.

In this experiment, we made an interesting discovery. As an animal model for surgical procedures, Diannan small-ear pig have incomparable advantages over small animals. First, the larger size of the pig makes the intraarticular anatomy easier to observe and obtain. Within the joint of the pig, it is easy to identify the various anatomical structures, such as the anterior and posterior cruciate ligaments, medial and lateral meniscus, and medial and lateral collateral ligaments. Second, surgical procedures are more convenient, such as infrapatellar fat pad that can easily be separated from the surrounding tissues. However, it is difficult to identify various tissues in the joints of small animals, and the acquisition amount is limited, which affects the experimental results. In conclusion, based on the results of this experiment, large animals like pigs are more recommended to be used for joint animal models. Some limitations in this study cannot be ignored. First, considering the economic and time cost with a preliminary exploration, only twenty-seven pigs were included in this study. To further explore the pathogenesis of OA, more animal specimens are needed in the future. Second, although the joint structure of Diannan small-ear pig1 was closer to human, and the pathological tissue was easier to manipulate and observe, the walking pattern of Diannan small-ear pig was different from humans. The next step will be to try to use animals that walk more like humans, such as monkeys. Third, morphologic and molecular biological changes in cartilage and its accessory structures should be included in a complete OA study. Unfortunately, this study was a preliminary exploration of a new animal model, and although relatively satisfactory results have been obtained, there was obviously a lack of in-depth study of synovial tissues. The follow-up research will be based on the experience of this study to explore the changes of synovial tissue in the process of OA in Diannan small-ear pig and to study related targeted treatment strategies.

## Conclusion

The Diannan small-ear pig can be used as an animal model for OA research because it is easy to obtain enough pathological tissues, and it is convenient for gross observation. Although two methods can induce obvious cartilage degeneration in pigs, an interesting and significant discovery of this study was that the chemical injection exhibited more and faster cartilage erosion, proteoglycan loss, and more serious synovial inflammation, which were all similar to those seen in human OA [29]. Intra-articular injection of papain is a better choice to induce OA due to less trauma and more efficient simulation of OA pathological processes. We have demonstrated that an antagonist of CXCR4, T140, decreased the levels of SDF-1. It prevented cartilage degeneration in OA guinea pigs through subcutaneous pumping [22]. Thus, our future work will be focused on the evaluation of the effect of SDF-1/CXCR4 axis blockage in OA Diannan small-ear pigs.

## Data Availability

The datasets used and/or analysed during the current study are available from the corresponding author on reasonable request.

## Abbreviations

OA: Osteoarthritis
ECM: Extracellular matrix
SDF-1: Stromal cell-derived factor-1
CXCR4: C-X-C chemokine receptor type 4
MMP: Matrix metalloproteinase
Col II: Collagen type II
ACAN: Aggrecan
ACL: Anterior cruciate ligament
PCL: Posterior cruciate ligament
MCL: Medial collateral ligament
MM: Medial meniscus
H&E: Hematoxylin
IgG: Immunoglobulin G
IOD: Integrated optical density
PVDF: Polyvinylidene difluoride
BSA: Bovine serum albumin
ANOVA: One-way analysis of variance
RT-qPCR: Reverse transcription quantitative polymerase chain reaction

## References

[1] Li L, Li Z, Li Y, Hu X, Zhang Y, Fan P (2020). Profiling of inflammatory mediators in the synovial fluid related to pain in knee osteoarthritis. BMC Musculoskelet Disord.

[2] Belluzzi E, Macchi V, Fontanella CG, Carniel EL, Olivotto E, Filardo G (2020). Infrapatellar fat pad gene expression and protein production in patients with and without Osteoarthritis. Int J Mol Sci.

[3] Englund M, Guermazi A, Lohmander SL (2009). The role of the meniscus in knee osteoarthritis: a cause or consequence?. Radiol Clin North Am.

[4] Stewart HL, Kawcak CE (2018). The importance of subchondral bone in the pathophysiology of Osteoarthritis. Front Vet Sci.

[5] Chen Y, Lin S, Sun Y, Guo J, Lu Y, Suen CW (2017). Attenuation of subchondral bone abnormal changes in osteoarthritis by inhibition of SDF-1 signaling. Osteoarthritis Cartilage.

[6] Lu W, Shi J, Zhang J, Lv Z, Guo F, Huang H (2016). CXCL12/CXCR4 axis regulates aggrecanase activation and cartilage degradation in a post-traumatic osteoarthritis rat model. Int J Mol Sci.

[7] Goudarzi R, Reid A, McDougall JJ (2018). Evaluation of the novel avocado/soybean unsaponifiable Arthrocen to alter joint pain and inflammation in a rat model of osteoarthritis. PLoS ONE.

[8] Lindström E, Rizoska B, Tunblad K, Edenius C, Bendele AM, Maul D (2018). The selective cathepsin K inhibitor MIV-711 attenuates joint pathology in experimental animal models of osteoarthritis. J Transl Med.

[9] Sandker MJ, Duque LF, Redout EM, Chan A, Que I, Löwik C (2017). Degradation, intra-articular retention and biocompatibility of monospheres composed of [PDLLA-PEG-PDLLA]-b-PLLA multi-block copolymers. Acta Biomater.

[10] Dubuc J, Girard C, Richard H, De Lasalle J, Laverty S (2018). Equine meniscal degeneration is associated with medial femorotibial osteoarthritis. Equine Vet J.

[11] Wang X, Li Y, Han R, He C, Wang G, Wang J (2014). Demineralized bone matrix combined bone marrow mesenchymal stem cells, bone morphogenetic protein-2 and transforming growth factor-β3 gene promoted pig cartilage defect repair. PLoS ONE.

[12] Wang Z, Zheng C, Zhong Y, He J, Cao X, Xia H (2017). Interleukin-17 Can Induce Osteoarthritis in Rabbit Knee Joints Similar to Hulth's Method. Biomed Res Int.

[13] Pomonis JD, Boulet JM, Gottshall SL, Phillips S, Sellers R, Bunton T (2005). Development and pharmacological characterization of a rat model of osteoarthritis pain. Pain.

[14] Alves AC, Albertini R, dos Santos SA, Leal-Junior EC, Santana E, Serra AJ (2014). Effect of low-level laser therapy on metalloproteinase MMP-2 and MMP-9 production and percentage of collagen types I and III in a papain cartilage injury model. Lasers Med Sci.

[15] Deng MW, Wei SJ, Yew TL, Lee PH, Yang TY, Chu HY (2015). Cell therapy with G-CSF-mobilized stem cells in a rat osteoarthritis model. Cell Transpl.

[16] Lynn SK, Kajaks T, Costigan PA (2008). The effect of internal and external foot rotation on the adduction moment and lateral-medial shear force at the knee during gait. J Sci Med Sport.

[17] Jeong DH, Ullah HMA, Goo MJ, Ghim SG, Hong IH, Kim AY (2018). Effects of oral glucosamine hydrochloride and mucopolysaccharide protein in a rabbit model of osteoarthritis. Int J Rheum Dis.

[18] Xu L, Peng Q, Xuan W, Feng X, Kong X, Zhang M (2016). Interleukin-29 enhances synovial inflammation and cartilage degradation in osteoarthritis. Mediators Inflamm.

[19] Kanbe K, Takagishi K, Chen Q (2002). Stimulation of matrix metalloprotease 3 release from human chondrocytes by the interaction of stromal cell-derived factor 1 and CXC chemokine receptor 4. Arthritis Rheum.

[20] Thomas NP, Wu WJ, Fleming BC, Wei F, Chen Q, Wei L (2017). Synovial inflammation plays a greater role in post-traumatic osteoarthritis compared to idiopathic osteoarthritis in the Hartley guinea pig knee. BMC Musculoskelet Disord.

[21] Kanbe K, Takemura T, Takeuchi K, Chen Q, Takagishi K, Inoue K (2004). Synovectomy reduces stromal-cell-derived factor-1 (SDF-1) which is involved in the destruction of cartilage in osteoarthritis and rheumatoid arthritis. J Bone Joint Surg Br.

[22] Wang K, Li Y, Han R, Cai G, He C, Wang G (2017). T140 blocks the SDF-1/CXCR4 signaling pathway and prevents cartilage degeneration in an osteoarthritis disease model. PLoS ONE.

[23] Eldridge S, Nalesso G, Ismail H, Vicente-Greco K, Kabouridis P, Ramachandran M (2016). Agrin mediates chondrocyte homeostasis and requires both LRP4 and α-dystroglycan to enhance cartilage formation in vitro and in vivo. Ann Rheum Dis.

[24] Wang D, Ren H, Li C, Liu F, Wang G, Shi F (2018). Orexin A prevents degradation of the articular matrixes in human primary chondrocyte culture. Mol Immunol.

[25] Yang CY, Chanalaris A, Troeberg L (2017). ADAMTS and ADAM metalloproteinases in osteoarthritis - looking beyond the 'usual suspects'. Osteoarthritis Cartilage.

[26] Chen LW, Liu FC, Hung LF, Huang CY, Lien SB, Lin LC, et al. Chondroprotective effects and mechanisms of dextromethorphan: repurposing antitussive medication for osteoarthritis treatment. Int J Mol Sci. 2018;19(3). 10.3390/ijms19030825 PMC5877686 29534535

[27] Hou SM, Hou CH, Liu JF (2017). CX3CL1 promotes MMP-3 production via the CX3CR1, c-Raf, MEK, ERK, and NF-κB signaling pathway in osteoarthritis synovial fibroblasts. Arthritis Res Ther.

[28] Motomura H, Seki S, Shiozawa S, Aikawa Y, Nogami M, Kimura T (2018). A selective c-Fos/AP-1 inhibitor prevents cartilage destruction and subsequent osteophyte formation. Biochem Biophys Res Commun.

[29] Leyh M, Seitz A, Dürselen L, Schaumburger J, Ignatius A, Grifka J (2014). Subchondral bone influences chondrogenic differentiation and collagen production of human bone marrow-derived mesenchymal stem cells and articular chondrocytes. Arthritis Res Ther.

